# SEPT12–NDC1 Complexes Are Required for Mammalian Spermiogenesis

**DOI:** 10.3390/ijms17111911

**Published:** 2016-11-16

**Authors:** Tsung-Hsuan Lai, Ying-Yu Wu, Ya-Yun Wang, Mei-Feng Chen, Pei Wang, Tsung-Ming Chen, Yi-No Wu, Han-Sun Chiang, Pao-Lin Kuo, Ying-Hung Lin

**Affiliations:** 1 Department of Obstetrics and Gynecology, Cathay General Hospital, Taipei 106, Taiwan; Joseph@cgh.org.tw; 2 School of Medicine, Fu Jen Catholic University, New Taipei City 242, Taiwan; 078575@mail.fju.edu.tw; 3 Institute of Systems Biology and Bioinformatics, National Central University, Jhongli City, Taoyuan Country 320, Taiwan; 4 Graduate Institute of Biomedical and Pharmaceutical Science, Fu Jen Catholic University, New Taipei City 242, Taiwan; l43219713@hotmail.com (Y.-Y.W.); 133838@mail.fju.edu.tw (Y.-N.W.); 053824@mail.fju.edu.tw (H.-S.C.); 5 Department of Chemistry, Fu Jen Catholic University, New Taipei City 242, Taiwan; vic0009@yahoo.com.tw; 6 Bone and Joint Research Center, Chang Gung Memorial Hospital, Taoyuan 333, Taiwan; mfchen0@gmail.com; 7 Department and Graduate Institute of Aquaculture, National Kaohsiung Marine University, Kaohsiung 811, Taiwan; tmtonychen@webmail.nkmu.edu.tw; 8 Department of Obstetrics & Gynecology, College of Medicine, National Cheng Kung University, Tainan 701, Taiwan; paolink@mail.ncku.edu.tw

**Keywords:** male infertility, SEPT12, NDC1

## Abstract

Male factor infertility accounts for approximately 50 percent of infertile couples. The male factor-related causes of intracytoplasmic sperm injection failure include the absence of sperm, immotile sperm, immature sperm, abnormally structured sperm, and sperm with nuclear damage. Our knockout and knock-in mice models demonstrated that SEPTIN12 (SEPT12) is vital for the formation of sperm morphological characteristics during spermiogenesis. In the clinical aspect, mutated SEPT12 in men results in oligozoospermia or teratozoospermia or both. Sperm with mutated SEPT12 revealed abnormal head and tail structures, decreased chromosomal condensation, and nuclear damage. Furthermore, several nuclear or nuclear membrane-related proteins have been identified as SEPT12 interactors through the yeast 2-hybrid system, including NDC1 transmembrane nucleoporin (NDC1). NDC1 is a major nuclear pore protein, and is critical for nuclear pore complex assembly and nuclear morphology maintenance in mammalian cells. Mutated NDC1 cause gametogenesis defects and skeletal malformations in mice, which were detected spontaneously in the A/J strain. In this study, we characterized the functional effects of SEPT12–NDC1 complexes during mammalian spermiogenesis. In mature human spermatozoa, SEPT12 and NDC1 are majorly colocalized in the centrosome regions; however, NDC1 is only slightly co-expressed with SEPT12 at the annulus of the sperm tail. In addition, SEPT12 interacts with NDC1 in the male germ cell line through coimmunoprecipitation. During murine spermiogenesis, we observed that NDC1 was located at the nuclear membrane of spermatids and at the necks of mature spermatozoa. In male germ cell lines, NDC1 overexpression restricted the localization of SEPT12 to the nucleus and repressed the filament formation of SEPT12. In mice sperm with mutated SEPT12, NDC1 dispersed around the manchette region of the sperm head and annulus, compared with concentrating at the sperm neck of wild-type sperm. These results indicate that SEPT12–NDC1 complexes are involved in mammalian spermiogenesis.

## 1. Introduction

### 1.1. Male Infertility

Between 2% and 12% of couples globally are affected by low fertility, and the cause in approximately half of these cases can be traced to the men [1]. Over the last 20 years, advanced intracytoplasmic sperm injection has been a technique used in the treatment of numerous subfertile men, who still produce small amounts of sperm that can be used for injection treatment [2]. Nevertheless, although intracytoplasmic sperm injection (ICSI) created a breakthrough in assisted reproduction, many infertile cases are still unable to achieve paternity, even when combined with testicular sperm extraction. The sperm-related causes of ICSI failure include immotile or immature sperm, sperm with structural defects, the absence of sperm, and sperm with premature chromosomal condensation or DNA damage [3].

### 1.2. SEPTINs (SEPTs) and Spermatogenesis

SEPTINs (SEPTs) are highly preserved polymerizing GTP-binding proteins that belong to the fourth component of the cytoskeleton [4]. SEPTs participate in membrane compartmentalization, cytoskeletal remodeling, cell polarity, and spermatogenesis through interaction with cytoskeletal proteins [5]. SEPTs are involved in the pathogenesis of various conditions, including Alzheimer disease, hereditary neuralgic amyotrophy, leukemia, ovarian tumors, breast cancer, and male infertility [5,6]. During mammalian spermiogenesis, SEPT4 is located at the annulus, which is a ring-like structure connecting the midpiece and principal piece of the flagellum, and is required to maintain annulus integration [7,8]. Clinically, most spermatozoa from asthenozoospermia patients lost the SEPT4 signal [7,9,10]. In our groups, SEPT12 was identified as a potential sterile gene by employing a cDNA microarray analysis of the testicular tissue, which determined that SEPT12 was expressed in postmeiotic male germ cells during mammalian spermiogenesis [11,12]. Furthermore, sperm from SEPT12-mutated mice exhibited unique morphological defects (e.g., immature sperm head, bent tail, premature chromosomal condensation, and nuclear damage) [13]. In humans, SEPT12 mutations in infertile men result in teratozoospermia and oligozoospermia [14,15,16]. In addition, several nuclear or nuclear membrane-related proteins were identified as SEPT12 interactors through the yeast 2-hybrid system; one of these interactors is NDC1 [17].

### 1.3. Nuclear Pore Complexes and NDC1

In eukaryotic cells, the nuclear envelope (NE) defines a boundary between the nucleus and cytoplasm and is composed of an outer and an inner nuclear membrane [18]. Outer and inner nuclear membranes are fused to form nuclear pore complexes (NPCs), which mediate the nucleocytoplasmic transport. The NPC comprises approximately 30 nucleoproteins (Nups), which can be subdivided into four classes: (1) transmembrane ring Nups; (2) core scaffold (inner and outer ring Nups); (3) linker Nups; and (4) nuclear Phe-Gly (FG) Nups [19,20]. NPCs are anchored to the NE through transmembrane ring Nups, which are composed of three proteins: nucleoporin 210 (NUP210), NDC1, and POM121 transmembrane nucleoporin (POM121) in vertebrate cells [20]. NDC1 and POM121 are critical for nuclear assembly, whereas NUP210 is a major molecule for efficient nuclear pore complex disassembly and NE breakdown [21,22,23]. Akiyama et al. indicated that mutated NDC1 (also called Transmembrane Protein 48; TMEM48) causes gametogenesis defects and skeletal malformations in mice, and this strain was spontaneously detected at The Jackson Laboratory in skeletal fusions with sterility (sks) in mice [24].

To better understand the molecular mechanism explaining the association of SEPT12 mutation with teratozoospermia, a yeast 2-hybrid approach was conducted, allowing the identification of NDC1 as a potential SEPT12 interactor. In this study, we observed that NDC1 was expressed around the NE in round spermatids and at the neck of mature spermatozoa. Ectopic overexpressed NDC1 restricted SEPT12 localization at the nucleus and repressed SEPT12 filament formation. In SEPT12-mutated spermatozoa, the location of NDC1 was disturbed. According to these results, we suggest that SEPT12–NDC1 complexes are involved in sperm head and tail formation during mammalian spermiogenesis.

## 2. Results

### 2.1. NDC1 as a SEPT12 Interactor

**Figure 1:**
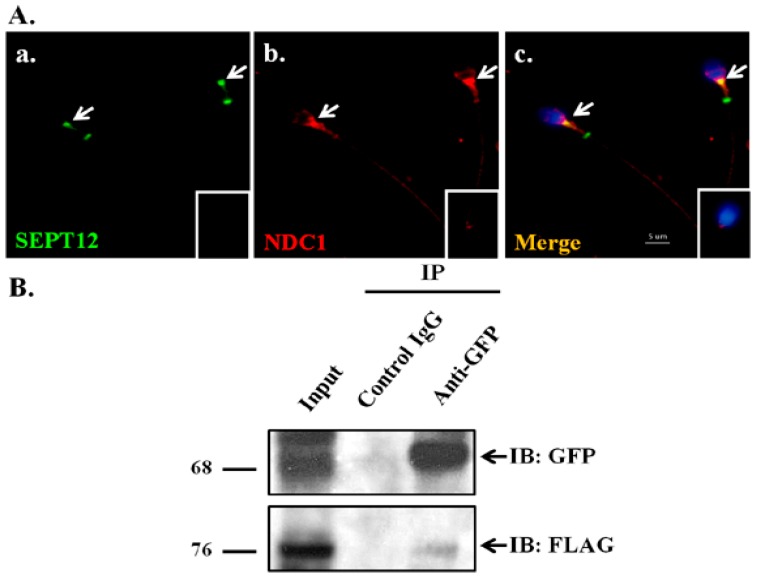
Colocalization and interaction between SEPT12 and NDC1. (**A**) Immunofluorescence detection in a mature human spermatozoon of (**a**) SEPT12; (**b**) NDC1; and (**c**) merged SEPT12 and NDC1. White arrows indicate the centrosome regions of the sperm. Insets reveal control IgG staining. Scale bar = 5 μm; (**B**) coimmunoprecipitation assay of FLAG-NDC1 and SEPT12-GFP. Lysates from transfected cells were immunoprecipitated with an anti-GFP antibody (Lane 3) or a nonspecific control IgG (Lane 2), followed by IB with an anti-GFP (indicated by black arrow) or anti-FLAG antibody (indicated by black arrow). An input protein (5%) was used as the control in IB of the transfected cell lysates (Lane 1).

In our previous study, SEPT12 mutations in human and mice spermatozoa were demonstrated to cause teratozoospermia (e.g., nuclear damage, premature chromosomal condensation, and abnormal morphologies of sperm heads and tails) [13,14,15,25]. NDC1 was identified as a SEPT12 interactor through the yeast 2-hybrid system by using a human testicular cDNA library [17]. In mice models, mutated NDC1 also affected mammalian spermatogenesis [25]. We suggest that mutated SEPT12 disturbed the spermiogenesis through NDC1. First, to test whether SEPT12 interacts with NDC1 in human male germ cells, we conducted an immunofluorescence assay (IFA) and coimmunoprecipitation assay (Co-IP). In mature human spermatozoa, SEPT12 and NDC1 are majorly colocalized in the centrosome regions; however, NDC1 is only slightly co-expressed with SEPT12 at the annulus of the sperm tail (Figure 1A). In mouse spermatozoa, colocalization patterns of SEPT12 and NDC1 are similar to those of human spermatozoa [26]. Second, NTERA-2 cl.D1 (NT2D1) cells, a pluripotent human testicular embryonal carcinoma cell line, were cotransfected with pFLAG-NDC1 and pGFP-SEPT12 vectors and then subjected to Co-IP with anti-GFP antibody. The Co-IP assays performed using the anti-FLAG antibody revealed that FLAG-NDC1 was pulled down with SEPT12-GFP (Figure 1B, Lane 3: Anti-GFP antibody). These results indicated that SEPT12 interacts with NDC1 in human male germ cells.

### 2.2. Dynamic Expression of NDC1 during Murine Spermiogenesis

**Figure 2:**
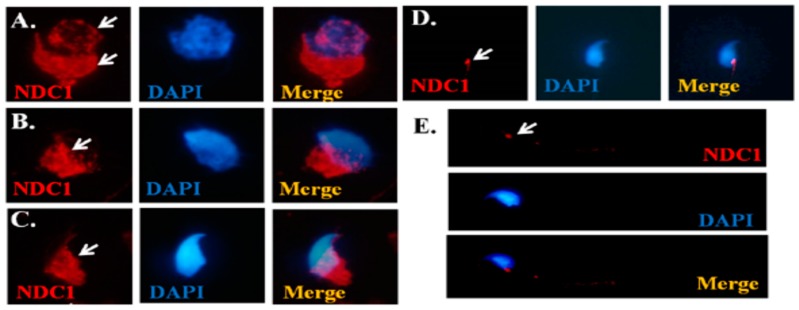
Localization of NDC1 during murine spermiogenesis. Immunofluorescence assay (IFA) results revealed multiple localizations of NDC1 signals: (**A**) round spermatids; (**B**,**C**) elongating spermatids; (**D**) elongated spermatids; and (**E**) mature spermatozoa. Anti-NDC1 antibody (“NDCI” panels), DAPI antibody (“DAPI” panels), and a combination of anti-NDC1 and DAPI antibodies (“Merge” panels) were used as stains. The arrows indicate NDC1 signals. NDC1: red; DAPI: blue. Magnification = 1000×.

To define the dynamic expression patterns of NDC1 during mouse spermiogenesis, the murine testicular germ cell populations were separated, and IFAs were performed. We determined that NDC1 was located around the nuclear membrane in the round spermatid stage (Figure 2A). Furthermore, in the elongating spermatid stage, NDC1 was expressed in the manchette regions of the developing sperm head (Figure 2B,C), and was finally localized in the neck region of mature spermatozoa (Figure 2D,E). These findings suggested that NDC1 is involved in sperm head and tail formation during murine spermiogenesis.

### 2.3. Effect of NDC1 on SEPT12 Localization and Filamental Structure in a Cell Model

**Figure 3:**
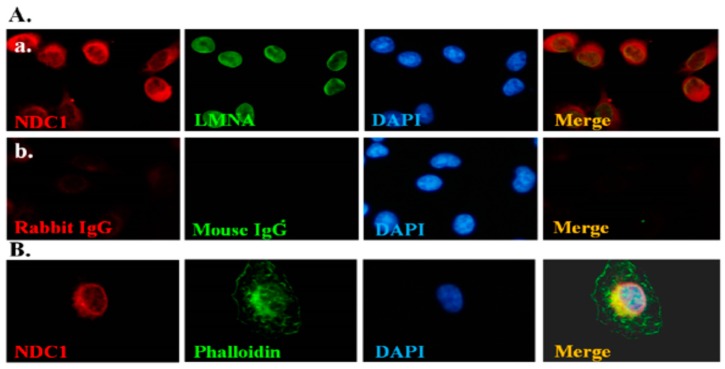
NDC1 location near the nuclear envelope in the male germ cell line. IFA results depicting the signals of (**A**) (**a**) FLAG-NDC1 using NDC1 (red), LAMNB1 (green), DAPI (blue), and a combination of NDC1, LAMN, and DAPI (“Merge” panel) stains; and (**b**) control IgG. Magnification = 400×. IFA results depicting the signals of (**B**) FLAG-NDC1 using NDC1 (red), Phalloidin (green), DAPI (blue), and a combination of NDC1, Phalloidin, and DAPI (“Merge” panel) stains. Magnification = 400×.

**Figure 4:**
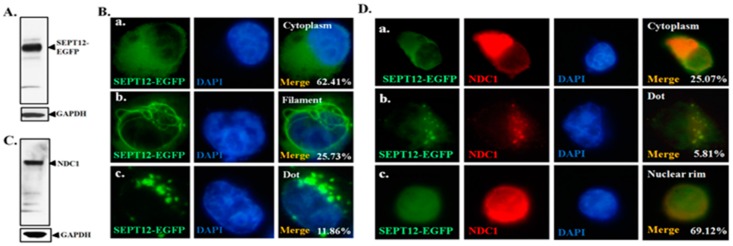
Effects of NDC1 overexpression on SEPT12 localization. (**A**) Western blotting of cells transfected with SEPT12-EGFP; (**B**) IFA results showing SEPT12-EGFP patterns: (**a**) localized at cytoplasm, (**b**) fibrous, or (**c**) dot-like; (**C**) Western blotting of cells with coexpressed SEPT12-EGFP and pFLAG-NDC1; (**D**) IFA results showing SEPT12-EGFP patterns: (**a**) localized at cytoplasm, (**b**) dot-like, or (**c**) localized at nuclear rim; (**B**,**D**) signals from EGFP protein (green), anti-FLAG antibody (red), DAPI (blue), and combined EGFP, anti-FLAG, and DAPI (“Merge” panels). Magnification = 1000×.

Figure 1 illustrates the interaction of NDC1 with SEPT12 and Figure 2 illustrates the expression of NDC1 during sperm head and tail formation; specifically, this NDC1 expression was similar to the SEPT12 patterns found previously [25]. Thus, we suggest that NDC1 regulates SEPT12 localization and polymerization structure. First, pFLAG-NDC1 was transfected into NT2D1 cells to determine whether NDC1 localizes at the nuclear membrane in a male germ cell line. NDC1 was located around the NE (Figure 3). The cells transfected with SEPT12 alone showed three typical patterns: (1) localized at cytoplasm (62.41%); (2) filament structure (25.73%); and (3) dot-like structure (11.86%) (Figure 4A,B). The expressional types of SEPT12 in the cell line are similar to the physiological patterns at the various stages of postmeiotic male germ cells (e.g., filament structure around the manchette structure of the elongating spermatid and dot-like structure located at the necks of mature sperm) [11]. However, after cotransfection with NDC1 and SEPT12, the NDC1 restricted the SEPT12 to the nucleus (69.12%) and repressed the formation of the filament structure (0%) (Figure 4C,D). This result indicated that NDC1 modulates the SEPT12 localization in the male germ cell line.

### 2.4. Effect of Mutated SEPT12 on NDC1 Localization In Vivo

**Figure 5:**
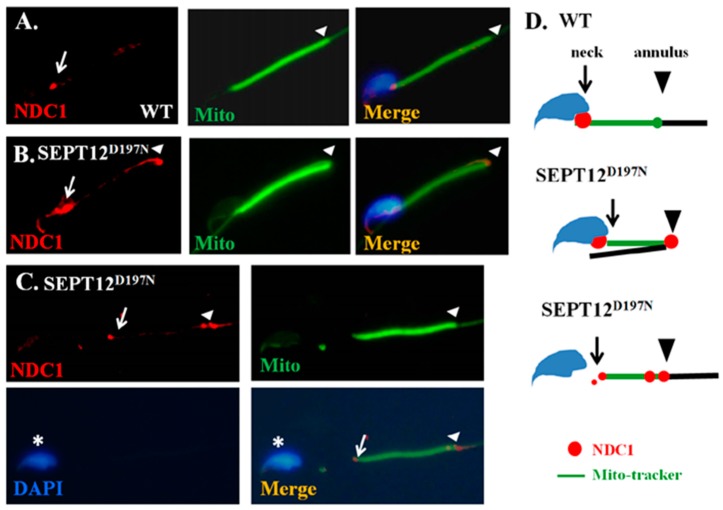
Localization of NDC1 in sperm from SEPT12D197N mice. IFA results showing multiple localizations of NDC1 signals in (**A**) wild-type sperm and (**B**,**C**) SEPT12D197N sperm. Anti-NDC1 antibody (“NDC1” panels), MitoTracker (“Mito” panels), DAPI (“DAPI” panel), and a combination of the anti-NDC1 antibody and MitoTracker (“Merge” panels) were used as stains. (**A**–**C**) NDC1: red; Mito: green; DAPI: blue. Arrows = sperm neck; Arrow head = annulus; * = sperm head. Magnification = 1000×; Scale bar = 5 μm; (**D**) a cartoon model of SEPT12D197N affecting NDC1 location. Blue area: Sperm-head; green-line: mito-tracker; black line: principal piece; arrow: sperm-neck; arrow head: annulus.

The SEPT12D197N (Asp197Asn; D197N) mutation has been isolated in infertile men with teratozoospermia and oligozoospermia [14,17]. In addition, knock-in mice with the SEPT12D197N mutation, which were used to mimic infertile men with SEPT12D197N, exhibited a high percentage of disorganized sperm annuli and necks [27]. To determine whether SEPT12 also affects NDC1 in vivo, IFA was performed in the sperm. In the bent-tail sperm isolated from the SEPT12D197N knock-in mice, NDC1 was dispersed around the manchette region of the sperm head and annulus, whereas in the wild-type mice, NDC1 was concentrated at the sperm neck and slightly at the annulus (Figure 5A,B). Furthermore, some of the sperm isolated from SEPT12D197N mice with a disabled neck were separated between the sperm head and tail, and also revealed substantial nonlocalized NDC1 signals at the sperm annulus (Figure 5C). These results indicated that loss of function because of SEPT12 mutations in vivo also affected dynamic localization of NDC1, and may affect precise sperm formation.

## 3. Discussion

### 3.1. SEPT12 Interaction with Nuclear and Nuclear-Related Proteins

We previously identified SEPT12 interactors, one of which was nuclear or nuclear membrane proteins. Notably, this protein group contains (1) NDC1 transmembrane nucleoproteins (NDC1); (2) sperm-associated antigen 4 proteins (SPAG4); and (3) protamine-2 proteins (PRM2) [17]. PRM2 and PRM1 are major sperm proteins involved in nuclear packing [28], whereas SPAG4 (SUN4) belongs to the SUN family. Additionally, SUN1 is one of the well-known nuclear membrane proteins, which links with LAMIN and the cytoplasmic cytoskeleton [29]. In our previous study, we found that SEPT12 interacts with SPAG4, but that the mutated SEPT12 disrupts this interaction [17]. NDC1 belongs to the transmembrane ring Nups, which are major proteins in NPCs anchored to the NE, and is vital for nuclear membrane assembly [21,22,23]. Moreover, the SUN protein, Msp3, in yeast cells controls NDC1 distribution and function in the nuclear membrane [30]. The present study is the first to reveal the association between SEPTs and the nucleoprotein NDC1.

### 3.2. Male Reproductive Roles of SEPT12 and NDC1

The sks in mice were spontaneously detected at The Jackson Laboratory [24]. Akiyama et al. indicated that NDC1 mutation induces abnormal splicing and exon 6 skipping in sks mice through linkage mapping and splicing assay [26]. Furthermore, they demonstrated that NDC1 were expressed in pachytene spermatocytes and following the sperm head in round and elongating spermatids [26]. In addition, mutated NDC1 induced meiosis arrest in pachytene spermatocytes and increased DNA double-strand breaks. In a previous study, we demonstrated that SEPT12 proteins were expressed at the sperm head during spermatid elongation, and at the neck and annulus of mature sperm [25]. Furthermore, sperm containing a mutated SEPT12 allele exhibited an immature sperm head, bent tail, premature chromosomal condensation, and nuclear damage [13]. During murine spermatogenesis, NDC1 was majorly expressed at the spermatocyte and the subsequent spermatid stages, whereas SEPT12 was specifically expressed at the postmeiotic male germ cell stages. Moreover, NDC1 and SEPT12 were co-expressed at the spermatid stage. In the present study, we observed dynamic expressions of NDC1 around the NE in round spermatids, and at the manchette region in elongating spermatids (Figure 2). During spermiogenesis, numerous proteins are involved in sperm morphogenesis [31,32]. After the final step, some proteins migrate to the sperm neck and sperm tail for other functions (e.g., SPAG4); others move into the residual body and are thrown out along with the excess cytoplasm. The results of NDC1 expression are similar to those of SEPT12 expression in our previous study, specifically in the manchette region of the sperm head and tail formation [25]. Thus, we suggest that NDC1 is a regulator of SEPT12 function during sperm head and tail formation.

### 3.3. Effect of Mutated SEPT12 on Cellular Localization of NDC1

During mammalian spermiogenesis, the precise regulation of the manchette dynamic is critical for sperm head formation [31,32]. As depicted in Figure 1 and Figure 2, the SEPT12 and NDC1 proteins in this study formed complexes, and expressed similar patterns at the manchette and neck region during terminal differentiation of male germ cells. In humans, the mutated SEPT12D197N (Asp197Asn), SEPT12T89M (Thr89Met), and SEPT12Del (c.474G/A induced truncated form) caused teratozoospermia and oligozoospermia [14,15,16]. To mimic the effect of SEPT12D197N in vivo, we generated knock-in mice [27], for which, as Figure 5 illustrates, the NDC1 patterns were irregular and dispersed around the manchette region of the sperm head and tail. This phenomenon may be attributed to the following: (1) the disrupted SEPT12 affects NDC1 localization or (2) the degradation of mutated SEPT12 affects the sperm formation resulting in the mislocalization of NDC1. In this study, we could not disregard the possibility of (2). We hypothesized that SEPT12–NDC1 dynamic localization and functionalism facilitate precise sperm head and tail formation and prevent abnormally shaped spermatozoa.

## 4. Materials and Methods

### 4.1. Immunofluorescence Assay

Human semen collection from fertile men (*n* = 3) for health evaluation was approved by the Institutional Review Board of Cathay General Hospital. More than 100 sperm were stained per man. The animal studies were approved by the Institutional Animal Care and Use Committee of Fu Jen Catholic University (A10153; 22 November 2012). Three knock-in mice (more than 50 sperm per mice) were evaluated. For an immunofluorescence assay (IFA), sperm and cells transfected with vectors were treated with 0.1% Triton X-100, washed twice with Tris-buffered saline (TBS), and subsequently incubated with a primary antibody (NDC1: sc-161929, Santa Cruz Biotechnology Inc., Santa Cruz, CA, USA; SEPT12:H00124404-B01P, Abnova, Taipei, Taiwan; LAMINB1: ab16048, Abcam, Cambridge, MA, USA) and secondary antibody (Alexa Fluor-568 donkey anti-goat IgG antibody: cat no. A-11057, Invitrogen, Carlsbad, CA, USA; Alexa Fluor-488 donkey anti-rabbit IgG antibody: cat no. A-21206, Invitrogen; Alexa Fluor-488 donkey anti-mouse IgG antibody; cat no. A-21202, Invitrogen). The procedure for IFA analysis was described in our previous study [33].

### 4.2. Cloning, Transfection, and Coimmunoprecipitation Assay

Human SEPT12 and NDC1 were amplified from a human RNA panel (Clontech, Mountain View, CA, USA) and cloned into pEGFP-N1 and pFLAG-CMV2 vectors, as described previously [33]. NT2D1 cells were transfected with the vectors through lipofection. Overexpression of NDC1 or co-transfection with SEPT12 was replicated three times and more than 100 cells were counted per assay. The cells lysates and the antibodies (Anti-GFP antibody: sc-9996, Santa Cruz Biotechnology Inc.; anti-FLAG antibody: F1804, Sigma-Aldrich, St. Louis, MO, USA; Anti-NDC1 antibody: sc-161929, Santa Cruz Biotechnology Inc.) were used in the Co-IP and IB assays, by employing the procedure described in our previous study [33].

### 4.3. Separation of the Murine Testicular Germ Cell Populations and Sperm Preparation

The spermatogenic cells isolated from mice (*n* = 3) were separated on the basis of the density of various germ cell types by using a centrifugal system, as described previously [33].

## 5. Conclusions

In this study, we demonstrated that SEPT12 binds with NDC1 and forms a complex during sperm head and tail formation. Overexpression of NDC1 affected the localization of SEPT12 in the cell. Moreover, mutated SEPT12 disturbed the localization of NDC1. Thus, we conclude that SEPT12–NDC1 function is involved in the morphogenesis of male germ cells.
